# Postmortem Interval and Diagnostic Performance of the Autopsy Methods

**DOI:** 10.1038/s41598-018-34436-1

**Published:** 2018-10-31

**Authors:** Juan Carlos Hurtado, Llorenç Quintó, Paola Castillo, Carla Carrilho, Fabiola Fernandes, Dercio Jordao, Lucilia Lovane, Mireia Navarro, Isaac Casas, Rosa Bene, Tacilta Nhampossa, Paula Santos Ritchie, Sónia Bandeira, Calvino Sambo, Valeria Chicamba, Sibone Mocumbi, Zara Jaze, Flora Mabota, Mamudo R. Ismail, Cesaltina Lorenzoni, Assucena Guisseve, Natalia Rakislova, Lorena Marimon, Natalia Castrejon, Ariadna Sanz, Anelsio Cossa, Inacio Mandomando, Khátia Munguambe, Maria Maixenchs, Carmen Muñoz-Almagro, Eusebio Macete, Pedro Alonso, Jordi Vila, Quique Bassat, Clara Menéndez, Miguel J. Martínez, Jaume Ordi

**Affiliations:** 1 ISGlobal, Hospital Clinic - Universitat de Barcelona, Barcelona, Spain; 2 Department of Microbiology, Hospital Clinic - Universitat de Barcelona, Barcelona, Spain; 3 Department of Pathology, Hospital Clinic - Universitat de Barcelona, Barcelona, Spain; 4 Department of Pathology, Maputo Central Hospital, Maputo, Mozambique; 5 Faculty of Medicine, Eduardo Mondlane University, Maputo, Mozambique; 6 Department of Medicine, Maputo Central Hospital, Maputo, Mozambique; 7 Centro de Investigação em Saúde de Manhiça, Maputo, Mozambique; 8 Department of Pediatrics, Maputo Central Hospital, Maputo, Mozambique; 9 Department of Gynecology and Obstetrics, Maputo Central Hospital, Maputo, Mozambique; 10 Department of Molecular Microbiology, University Hospital Sant Joan de Déu (University of Barcelona), Barcelona, Spain; 11 Consorcio de Investigación Biomédica en Red de Epidemiología y Salud Pública (CIBERESP), Madrid, Spain; 12 Faculty of Medicine, Univesitat Internacional de Catalunya, Barcelona, Spain; 13 ICREA, Catalan Institution for Research and Advanced Studies, Pg. Lluís Companys 23, 08010 Barcelona, Spain; 14 Pediatric Infectious Diseases Unit, Pediatrics Department, Hospital Sant Joan de Déu (University of Barcelona), Barcelona, Spain

## Abstract

Postmortem studies, including the complete diagnostic autopsy (CDA) and the minimally invasive autopsy (MIA), an innovative approach to post-mortem sampling and cause of death investigation, are commonly performed within 24 hours after death because the quality of the tissues deteriorates over time. This short timeframe may hamper the feasibility of the procedure. In this study, we compared the diagnostic performance of the two postmortem procedures when carried out earlier and later than 24 hours after death, as well as the impact of increasing postmortem intervals (PMIs) on the results of the microbiological tests in a series of 282 coupled MIA/CDA procedures performed at the Maputo Central Hospital in Mozambique between 2013 and 2015. 214 procedures were conducted within 24 hours of death (early autopsies), and 68 after 24 hours of death (late autopsies). No significant differences were observed in the number of non-conclusive diagnoses (2/214 [1%] vs. 1/68 [1%] p = 0.5645 for the CDA; 27/214 [13%] vs. 5/68 [7%] p = 0.2332 for the MIA). However, increasing PMIs were associated with a raise in the number of bacteria identified (rate: 1.014 per hour [95%CI: 1.002–1.026]; p = 0.0228). This increase was mainly due to rising numbers of bacteria of the *Enterobacteriaceae* family and *Pseudomonas* genus strains. Thus, performing MIA or CDA more than 24 hours after death can still render reliable diagnostic results, not only for non-infectious conditions but also for many infectious diseases, although, the contribution of *Enterobacteriaceae* and *Pseudomonas spp*. as etiological agents of infections leading to death may be overestimated.

## Introduction

The minimally invasive autopsy (MIA) is a postmortem diagnostic method based on obtaining samples of body fluids and key organs using needle biopsies. Such an approach has been designed as a potential alternative of the complete diagnostic autopsy (CDA)^1^ in resource limited settings. The MIA requires lower levels of expertise and infrastructures than the CDA^1^ and there are data showing a high level of interest in knowing cause of death and potential acceptability in low- and middle-income countries^2^. Moreover, the use of needles without manipulation of the organs allows collecting samples for microbiology analysis with lower risk of contamination compared with CDA. The procedure has recently been validated in Mozambique in all age groups, including stillbirths, neonates, children, adults and maternal deaths^3–6^. In this validation analysis, the concordance with the CDA, the gold standard for cause of death determination, was moderate to good, and the MIA reliably recognized most infectious diseases and cancers causing death. This new method has raised high expectations among global health researchers because it may provide more robust explanations for the cause of death than other currently used approaches^7–9^.

To deploy such methods to study mortality in low- and middle-income countries, we need to understand the timeframe limits whereby the results of a MIA are adequate, which have not been previously defined. However, data evaluating the possible effects of the postmortem interval (PMI) in the diagnostic yield of the MIA are scarce. In the clinical routine, the CDA is generally conducted within 24 hours after death^10,11^, because the quality of the tissues, and consequently, the quality of the results obtained, deteriorates over time due to autolysis, and other decay processes that affect the body^12^. One of these phenomena is the active participation of microorganisms in the decomposition of bodies. Temperature and anaerobic conditions have been proposed as major factors driving the decomposition process^13,14^. The bacterial translocation, a phenomenon that rapidly initiates following death and by which viable bacteria originating principally from the intestinal microbiota migrate to other organs, may pose challenges when trying to ascertain the contribution of detected microorganisms to the fatal event. Indeed, judging whether a potentially pathogenic microorganism is the expression of a true ante-mortem bacterial infection or a false-positive result related to events occurring after death is not straightforward^15,16^. Understandingly, general consensus recommends conducting any postmortem evaluation as soon as possible following death, once the family consent has been obtained. However, such short timeframes may hamper the feasibility of the MIA procedure, particularly when implemented in health facilities from rural areas where many deaths occur at home and it may take some time to reach the health facility.

When designing the MIA protocol, a period of 24 hours after death was established as the maximum “ideal time” to perform the procedure. However, due to a variety of circumstances, some of the autopsies were performed after this timeframe. In this study, we aimed to evaluate the impact of the PMI on the diagnostic performance of the MIA and the CDA and provide information on the timeframe limits in which the results of a postmortem evaluation may be adequate.

## Methods

### Study setting and design

This analysis derives from the CaDMIA project, a validation study of the MIA vs. the CDA in different age groups. The study was conducted at the Department of Pathology of the Maputo Central Hospital, a 1500-bed government-funded tertiary health care center, in collaboration with the departments of medicine, obstetrics and gynecology, and pediatrics. The study received the approval of the National Bioethics Committee of Mozambique (Ref. 342/CNBS/13) and the Clinical Research Ethics Committee of the Hospital Clinic of Barcelona in Spain (Ref: 2013/8677). All research was performed in accordance with relevant guidelines and regulations of the Montreal Statement on Research Integrity in Cross-Boundary Research Collaborations, and the Singapore Statement on Research Integrity.

Deaths of all ages having occurred within the hospital premises from November 2013 to March 2015 were submitted to coupled MIA and CDA procedures. Eligible criteria for case recruitment were: 1) an autopsy requested by the clinician as part of the medical evaluation of the patient, and 2) informed consent to perform the autopsy given by the relatives. Accidental or traumatic deaths were excluded.

### Autopsy procedures

Detailed MIA pathological and microbiological methods have been reported elsewhere^1,3–6^. In brief, the procedure included an initial disinfection of the surface of the body followed by the collection of blood and cerebrospinal fluid (CSF), aiming to collect ~10–15 mL of each fluid, and puncture of solid organs (liver, lungs, and central nervous system [CNS]) using biopsy needles (14G-16G), for microbiological and pathological analysis. In addition, punctures aiming to obtain heart, spleen, kidneys and, in maternal deaths, uterus, were performed for pathology examination.

Immediately after the MIA, the CDA procedure was conducted by a second pathologist not involved with the MIA and following a standardized protocol for adult deaths^17^ or pediatric and perinatal deaths^18^. Histological and microbiological samples were obtained from the same viscera collected in the MIA and CDA, as well as from any grossly identified lesions. Time of death and start of MIA and CDA procedures were recorded for all cases.

### Histological and microbiological analyses

The histological evaluation included staining with hematoxylin and eosin in all samples and additional histochemical and/or immunohistochemical staining whenever needed to reach a diagnosis^1,3–6^. All microbiological methods, that included both culture and molecular tests, have been reported in detail elsewhere^15,19^. Microbiological cultures were performed only with the fluids and tissues obtained in the MIA. Briefly, 5–10 mL of blood were inoculated into aerobic blood culture bottles and incubated into the BACTEC system (Beckton Dickinson, MD, USA). Positive blood cultures were subjected to Gram stain and sub-cultured in agar plates according to the morphology observed under the microscope. CSF samples were sub-cultured into blood, chocolate and MacConkey agar plates, because between all these culture media allow the recovery of the maximum amount of cultivable microorganisms with medical interest. Tissue samples from CNS, liver, lung and uterus were inoculated into thioglycolate broth-containing tubes, a multipurpose, enriched, differential medium and incubated at 37 °C. If signs of bacterial or fungal growth were observed, Gram stain and sub-culture into appropriate agar plates for the recovery of bacteria were performed; if yeasts were observed in the gram stain or if fungal filamentous growth were observed, sub-cultured into appropriate media was performed. Identification of the isolates was performed by standard biochemical techniques at the laboratory of the Manhiça Health Research Center (CISM). The strains were kept at −80 °C until shipment to the Hospital Clinic of Barcelona for final identification by matrix assisted laser desorption/ionization (MALDI-TOF, Bruker Daltonik GmbH, Bremen, Germany).

Molecular analyses were performed both in body fluids and tissues obtained in the MIA and in tissues obtained in the CDA. For these analyses, plasma and CSF aliquots, as well as tissue samples, were stored at −80 °C until testing. All tissue samples obtained through MIA and CDA procedures were homogenized using a handheld rotor-stator homogenizer (TissueRuptor, Qiagen) and disposable probes for each tissue sample processed. Nucleic acid extractions (DNA + RNA) were performed using a semi-automated system (Qiacube, Qiagen). For formalin-fixed paraffin embedded (FFPE) tissues, three thick sections of 10 μm were prepared. The microtome blade was replaced after each case to avoid cross-contamination. Nucleic acid extraction included overnight proteinase K digestion and DNA isolation using a commercially available kit (QIAamp DNA minikit, Qiagen) according to the manufacturer’s instructions. DNA from whole blood collected in filter paper was subjected to DNA extraction using PurelinkTM Genomic DNA Kit (Invitrogen, Waltham MA, USA).

We performed generic PCRs (16S rRNA PCR and 18S rRNA-ITS PCR) and specific PCR (e.g. cytomegalovirus real-time PCR, *Toxoplasma gondii* real time PCR, *Pneumocystis jirovecii* real time PCR, *Mycobacterium tuberculosis* real time PCR, multiplex respiratory virus RT-PCR, *P*. *falciparum* real time PCR)^18^. All sequencing reactions from amplicons obtained by either the 16S rRNA PCR or the 18S rRNA-ITS PCR were performed by the Sanger method. Pathogen identification was performed by comparison of the sequences obtained with those present in the GenBank using the BLAST algorithm (http://blast.ncbi.nlm.nih.gov/Blast.cgi).

Two scales ranging from 0 to 4 were developed to grade the strength of the evidence of the findings; one based on the severity of the pathological findings and the other on the pathogenicity of each individual microorganism, the number of organs (one or more organs) and the type of techniques (classic cultures, molecular tests or both) in which the microorganism was identified^3–6^. Microorganisms were categorized as causing true infection or as contaminants based on criteria previously reported by our group, which varied slightly for adults, children, neonates and stillborn babies^3–6^. Briefly, these criteria comprised the type of microorganism, the number of organs involved, the presence or absence of pathologic findings associated with the infection, and the clinical data (for the CDA). The true infection category included infections causing death and infections present during life as associated conditions. Any other microorganism identified not fulfilling these criteria were classified as contaminant microorganisms.

### Cause of death determination

Once all the analyses of the MIA samples had been completed, a panel composed of a pathologist, a microbiologist, and a clinician with expertise in infectious diseases and epidemiology evaluated all the pathological and microbiological results and assigned the MIA diagnosis, following a methodology previously descibed^3–6^.

Whenever identified, a chain of conditions leading to death (up to four) was recorded following the most probable chronological sequence of events^1,3–6^. The direct cause of death was considered as the main cause of death (e.g. bacterial pneumonia in a toddler with malnutrition, or lymphoma in an HIV-infected patient), only in the group of maternal death we considered the underlying disease as cause of death. Finally, other conditions or concomitant infections contributing to death but not related to the chain of events leading to death were considered as other significant conditions.

After a washout period (minimum 3 months, range 3–6 months), and blindly to the MIA diagnosis, the same team analyzed the samples of the CDA following the same approach used for the analysis of the MIA samples, with the only exception that tissues obtained during CDA were not routinely cultured, and only molecular methods were used to investigate pathogens. The panel evaluating the CDA integrated all the findings including not only the pathological and microbiological results, but also the clinical information and the macroscopic data.

Using a combination of the strength of the evidence of the histological and the microbiological findings, a category was assigned to the certainty of the cause of death attribution of the MIA diagnosis and the CDA diagnosis. These categories included no diagnosis and diagnosis of low, moderate, high, and very high certainty^3–6^.

The CDA diagnosis was considered the “gold standard”. Diagnoses were grouped into infectious diseases, non-infectious diseases and non-conclusive.

### Time lapse between death and postmortem procedures and definition of the study groups

The day and time of death (with a precision of minutes) as well as the exact day and time when the MIA procedure started were recorded for each case. The PMI was defined as the time from death to the MIA procedure. For stillborn babies in whom the precise time of death was not clearly defined, the PMI was defined as the time from delivery to the MIA. All cases were classified in two groups: a) cases in which the autopsy procedures started within the first 24 hours after death (“early autopsy” group); and b) all cases in which the autopsy procedures started after the first 24 hours after death (“late autopsy” group). The microorganisms identified, as well as the technique in which they were identified were correlated with the PMI. The bodies were kept under refrigeration from death until the postmortem procedure.

### Statistical methods and definitions

We compared the differences in the distribution of causes of death and the percentage of inconclusive cases observed in early and late autopsies. The differences were assessed using the Chi-square test or Fisher’s exact test. The differences in levels of strength and certainty between early and late MIAs were analyzed using the Student’s t-test for independent groups and Fisher’s exact test respectively. The coincidence between the MIA and the CDA diagnoses was evaluated, by comparing the ICD-10 codes of the main diagnosis^3–6^. As the ICD-10 system classifies diagnoses into nested classes of different hierarchical levels in which diseases or conditions are organized in chapters, blocks, and 3-character categories, a coincidence was classified as (i) perfect; (ii) moderate; or (iii) low^20^. When the MIA and the CDA diagnoses were in different chapters, the coincidence was classified as “none”.

For the microbiological tests, we analyzed both for the MIA (cultures and molecular tests) and the CDA (only molecular tests) the association between the microorganisms identified and the elapsed time after death. The sample performance was assessed using the following indicators: number of tests and number of tests with a positive result (detection of one or multiple microorganisms in a culture or in a molecular test). The positive results were divided into positive results due to microorganisms considered as contaminants (microorganism considered as associated with translocation and not causing disease) and positive results due to microorganisms considered as responsible of true infections (detection of microorganisms considered as associated with disease).

The analysis was stratified by the type of microbiological test used to identify the pathogen (culture or molecular test). The number of tests with positive results was analyzed as count data potentially with excess zeros, since the number of tests performed was not the same for all cases. Hence, zero inflated Poisson regression models (ZIP) or standard Poisson regression as appropriate according to the Vuong test^21^ were estimated using the number of tests as inflation factor and standard errors clustered at case level, relaxing the usual requirement that the observations be independent. The ZIP models were essentially two-stage models in which the probability of being tested was estimated first, followed by the estimation of Poisson regression among cases with a nonzero probability of being tested.

The concordance between MIA and CDA was assessed by the Cohen’s Kappa statistic and differences were assessed based on Student’s t distribution of 1000 bootstrap replications of paired differences for comparing correlated kappa coefficients^22^.

Statistical analyses were performed using Stata version 14.1 (Stata, College Station, TX, USA)^23^.

### Ethics approval and consent to participate

The study protocol received approval of the National Mozambican Ethics Committee (ref.342/CNBS/13) and the Ethics Committee of the Hospital Clinic of Barcelona (Spain; approved, File 2013/8677). MIA and CDA procedures were only conducted after verbal informed consent was provided by the relatives.

## Results

Coupled MIA and CDA procedures were performed in 282 individuals including 18 stillborn babies, 41 neonates, 54 children, 57 maternal deaths and 112 other adults older than 15 years.

Two hundred fourteen (75.9%) MIA/CDA procedures were conducted within the first 24 hours (early autopsies) and 68 (24.1%) more than 24 hours after death (late autopsies). In the early autopsy group, the median time after death was 14 hours (range 4–24 hours). In this group 12 (5.6%) cases were stillborn babies, 29 (13.6%) were neonates, 33 (15.4%) were children, 37 (17.3%) were women of childbearing age, and finally 103 (48.1%) were other adults. In the group of late autopsies, the median time after death was 27 hours (range 24–65 hours). Of them, 6 (8.9%) were stillborn babies, 12 (17.6%) were neonates, 21 (30.9%) were children, 20 (29.4%) were maternal deaths, and 9 (13.2%) were other adults. In this group the procedures were performed between 24 and 36 hours after death in 52 cases (76.5%), and after 36 hours or more after death (range 36–64 hours) in 16 cases (23.5%).

Table 1 shows the causes of death grouped by main categories (i.e. infectious vs. non-infectious, vs. no diagnosis) identified at the CDA (gold standard) in the different groups of patients. No differences were observed either in terms of diagnostic distribution between the early autopsies and the late autopsies or in the number of cases with non-conclusive diagnosis.

**Table 1:** Causes of death identified at the complete diagnostic autopsy (gold standard) in the different groups of patients in the early autopsy group (performed within 24 hours of death) and the late autopsy group (performed more than 24 hours after death).

	Time from death to autopsy procedures			p-value^*^
	Early autopsies (n = 214)	Late autopsies (n = 68)	Total (n = 282)	
Stillbirths				0.1787
Infectious diseases	1 (8%)	3 (50%)	4 (22%)	
Non-infectious diseases	9 (75%)	3 (50%)	12 (67%)	
Non-conclusive	2 (17%)	0 (0%)	2 (11%)	
*Total*	12 (*100%*)	6 (*100%*)	18 (*100%*)	
Neonates				0.1650
Infectious diseases	17 (59%)	10 (83%)	27 (66%)	
Non-infectious diseases	12 (41%)	2 (17%)	14 (34%)	
Non-conclusive	0 (0%)	0 (0%)	0 (0%)	
*Total*	29 (*100%*)	12 (*100%*)	41 (*100%*)	
Children				0.3291
Infectious diseases	24 (73%)	18 (86%)	42 (78%)	
Non-infectious diseases	9 (27%)	3 (14%)	12 (22%)	
Non-conclusive	0 (0%)	0 (0%)	0 (0%)	
*Total*	33 (*100%*)	*21* (*100%*)	54 (*100%*)	
Maternal deaths				0.4466
Infectious diseases	18 (49%)	8 (40%)	26 (45%)	
Non-infectious diseases	19 (51%)	11 (55%)	30 (53%)	
Non-conclusive	0 (0%)	1 (5%)	1 (2%)	
*Total*	37 (*100%*)	20 (*100%*)	57 (*100%*)	
Other adults				0.7132
Infectious diseases	74 (72%)	6 (67%)	80 (71%)	
Other Non-infectious diseases	29 (28%)	3 (33%)	32 (29%)	
Non-conclusive	0 (0%)	0 (0%)	0 (0%)	
*Total*	103 (*100%*)	9 (*100%*)	112 (*100%*)	

The data are presented as absolute numbers and (percentages of the column).

^*^Fisher’s exact test.

### Level of strength of the findings, level of certainty of the diagnoses and coincidence between the mia and the cda

No significant differences were found in the mean level of strength of the pathological and microbiological findings of the MIA and the CDA between early and late autopsy groups, with the only exception of a slightly lower level of strength of the pathological findings in the CDA in the late autopsy group (3.6 in the early, vs. 3.3 in the late, p = 0.0155) (see Supplementary Information Table 1).

Table 2 shows the level of certainty of the MIA and the CDA diagnoses in the early and late autopsy groups. No significant differences were identified between the two groups.

**Table 2:** Level of certainty of the MIA and the CDA diagnoses in the early (performed within 24 hours of death) and the late autopsy groups (performed more than 24 hours after death).

Level of certainty of the cause of death	Time from death to the postmortem procedures		p-value
	Early autopsies (n = 214) n (%)	Late autopsies (n = 68) n (%)	
Minimally invasive autopsy			0.1871*
Very high	74 (35%)	19 (28%)	
High	52 (24%)	17 (25%)	
Moderate	36 (17%)	20 (29%)	
Low	25 (12%)	7 (10%)	
No diagnosis	27 (13%)	5 (7%)	
Complete diagnostic autopsy			0.8169^#^
Very high	93 (43%)	26 (38%)	
High	80 (37%)	28 (41%)	
Moderate	29 (14%)	11 (16%)	
Low	10 (5%)	2 (3%)	
No diagnosis	2 (1%)	1 (1%)	

*Chi-square test; ^#^Fisher’s exact test.

Table 3 shows the concordance between the MIA and the CDA diagnoses in early and late autopsy groups. No differences were observed in any of the age groups.

**Table 3:** Concordance between the MIA and the CDA in the early (performed within 24 hours after death) and the late autopsy groups (performed more than 24 hours after death).

Group	Time from death to the postmortem procedures				p-value^†^
	Early autopsies (n = 214)		Late autopsies (n = 68)		
	Agreement (%)*	Kappa value^#^	Agreement (%)*	Kappa value^#^	
Stillbirths	10/12 (83%)	0.7576	5/6 (83%)	0.7692	0.9698
Neonates	18/29 (62%)	0.3794	10/12 (83%)	0.4419	0.8618
Children	28/33 (85%)	0.6429	20/21 (95%)	0.8372	0.4583
Maternal deaths	24/37 (65%)	0.4025	15/20 (75%)	0.6183	0.2376
Other adults	89/103 (86%)	0.7274	8/9 (89%)	0.7857	0.7679

Number of cases classified in the same disease group by MIA and CDA; ^#^Concordance between the MIA and the CDA; ^†^Based on Student’s t distribution of 1000 bootstrap replications of paired differences

### Relationship between the pmi and identification of microorganisms

**Figure 1:**
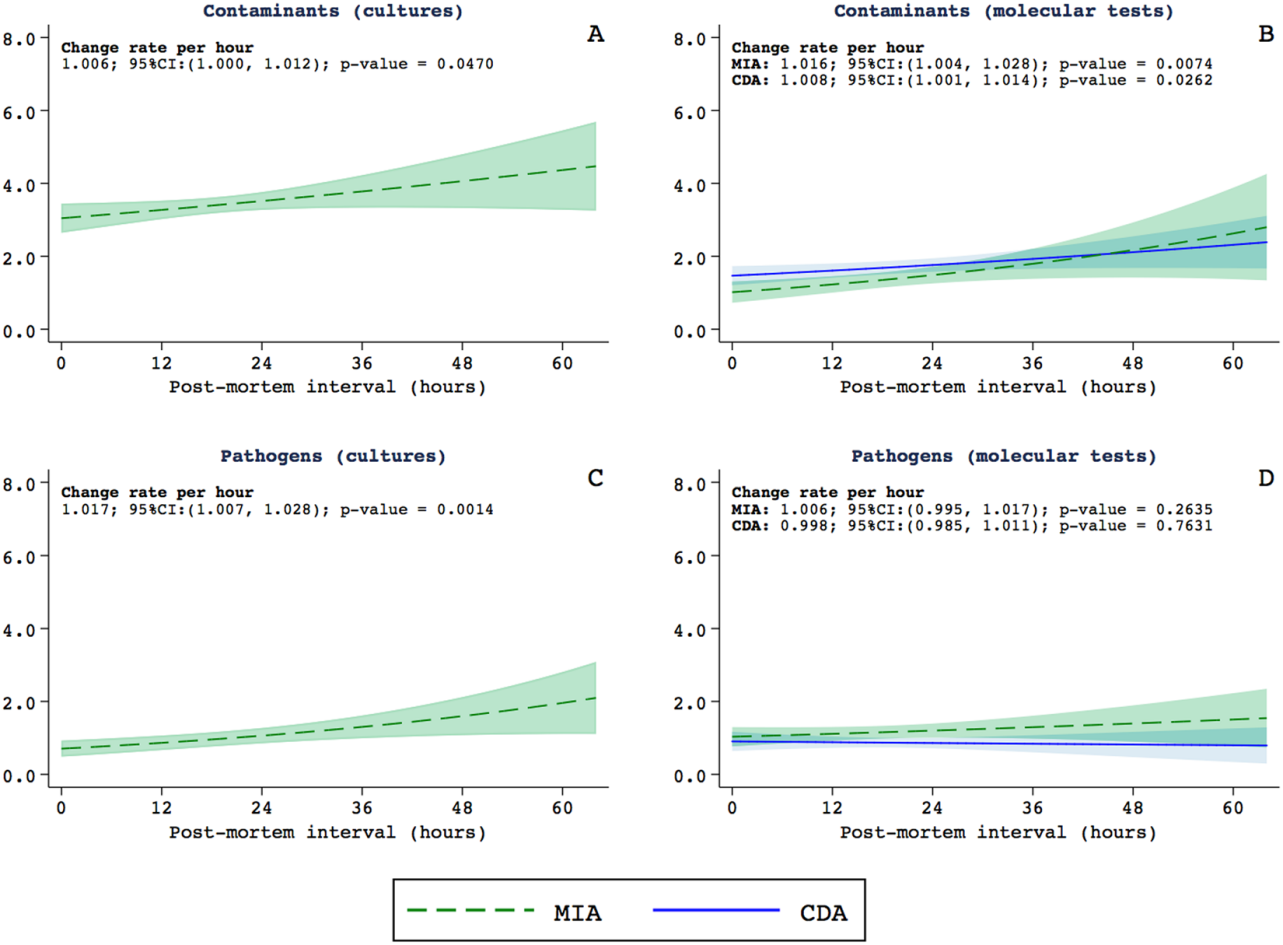
Relationship between the estimated rates of microorganisms identified and the postmortem interval. Microorganisms classified as contaminants are shown in (**A**,**B**) and the microorganisms considered as associated with true infections in (**C**,**D**). The estimations for the conventional cultures are shown in graphics (**A**,**C**) and those of the molecular tests in graphics (**B**,**D**). For the molecular tests, the graphics include the estimations for the minimally invasive autopsy (MIA, in green) and the complete diagnostic autopsy (CDA, in blue).

Figure 1 shows the relationship between the estimated rates of microorganisms identified and the PMI. The list of microorganisms identified in this study is shown in Supplementary Table 2. The results of the microorganisms classified as contaminants are presented in Fig. 1A,B, and those of the microorganisms considered as associated with true infections (either as direct causes of death, involved in the chain of events leading to death or present during life as associated conditions) in Fig. 1C,D. The estimations for the conventional cultures are shown in graphics 1A and 1C and those of the molecular tests in graphics 1B and 1D. For the molecular tests, the graphics include the estimations for the MIA (in green) and the CDA (in blue). Long PMI were associated with higher rates of contaminants in the conventional cultures and in the molecular tests (Fig. 1A,B respectively). For the microorganisms considered as associated with true infections the PMI was only associated with a higher rate of detection in conventional cultures but not in the molecular tests (Fig. 1C,D respectively). This association was observed in the samples of the cerebrospinal fluid (p = 0.0240), the brain (p < 0.0001), the lungs (p = 0.0001), and the uterus (p = 0.0447).

**Figure 2:**
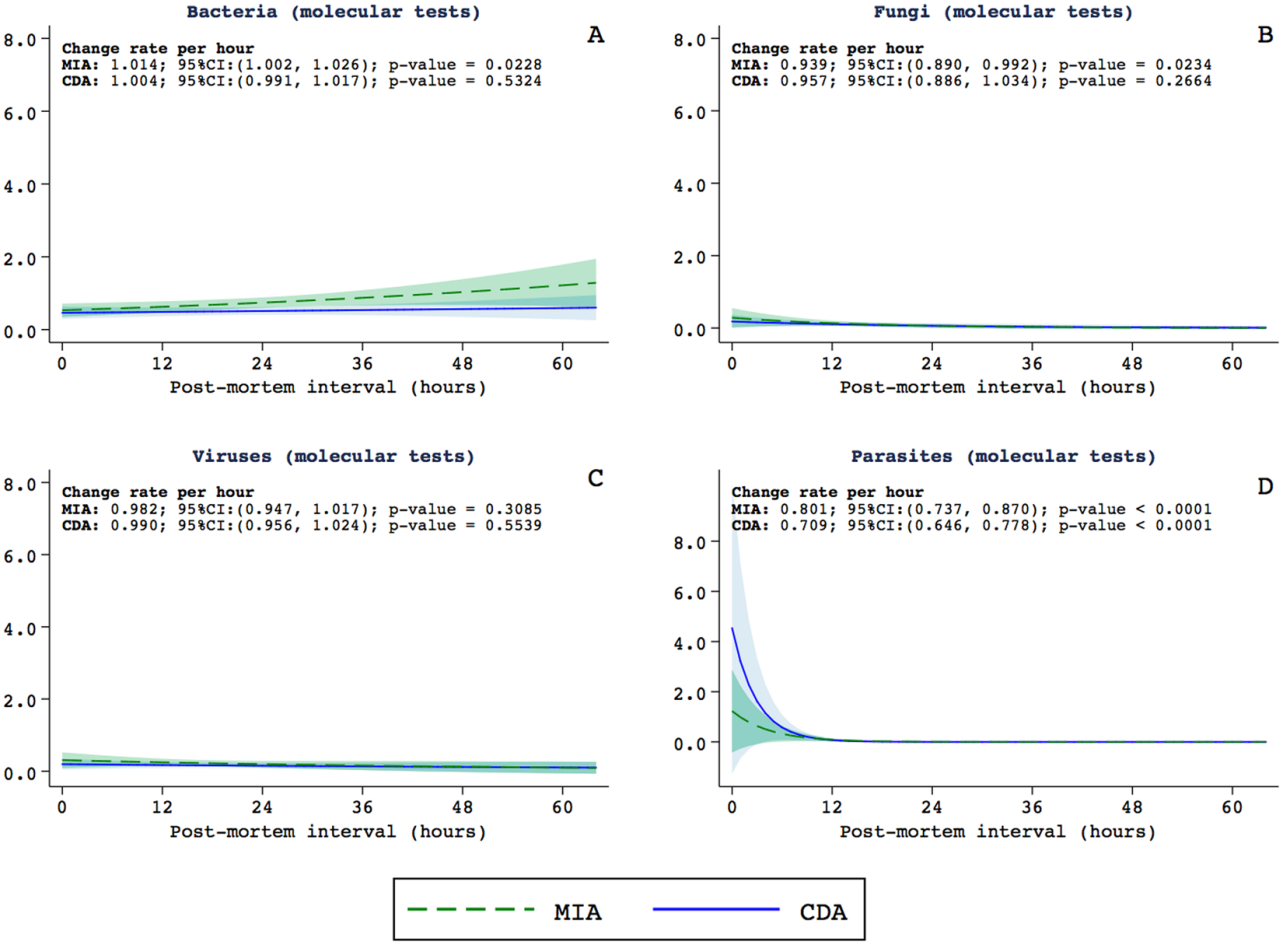
Relationship between the postmortem interval and the estimated detection rates of bacteria (**A**), fungi (**B**), viruses (**C**) and parasites (**D**) identified in the molecular tests. The graphics include the estimations for the minimally invasive autopsy (MIA, in green) and the complete diagnostic autopsy (CDA, in blue).

Figure 2 shows the relationship between the PMI and the estimated detection rates of bacteria, fungi, viruses and parasites identified in the molecular tests. Long PMI was associated with high detection of bacteria in the MIA samples. No relationship between the PMI and estimated detection rates of viruses (Fig. 2C). There was a statistically significant decrease in the identification of fungi and parasites in cases with the PMI longer than 12 hours (Fig. 2B,D).

**Figure 3:**
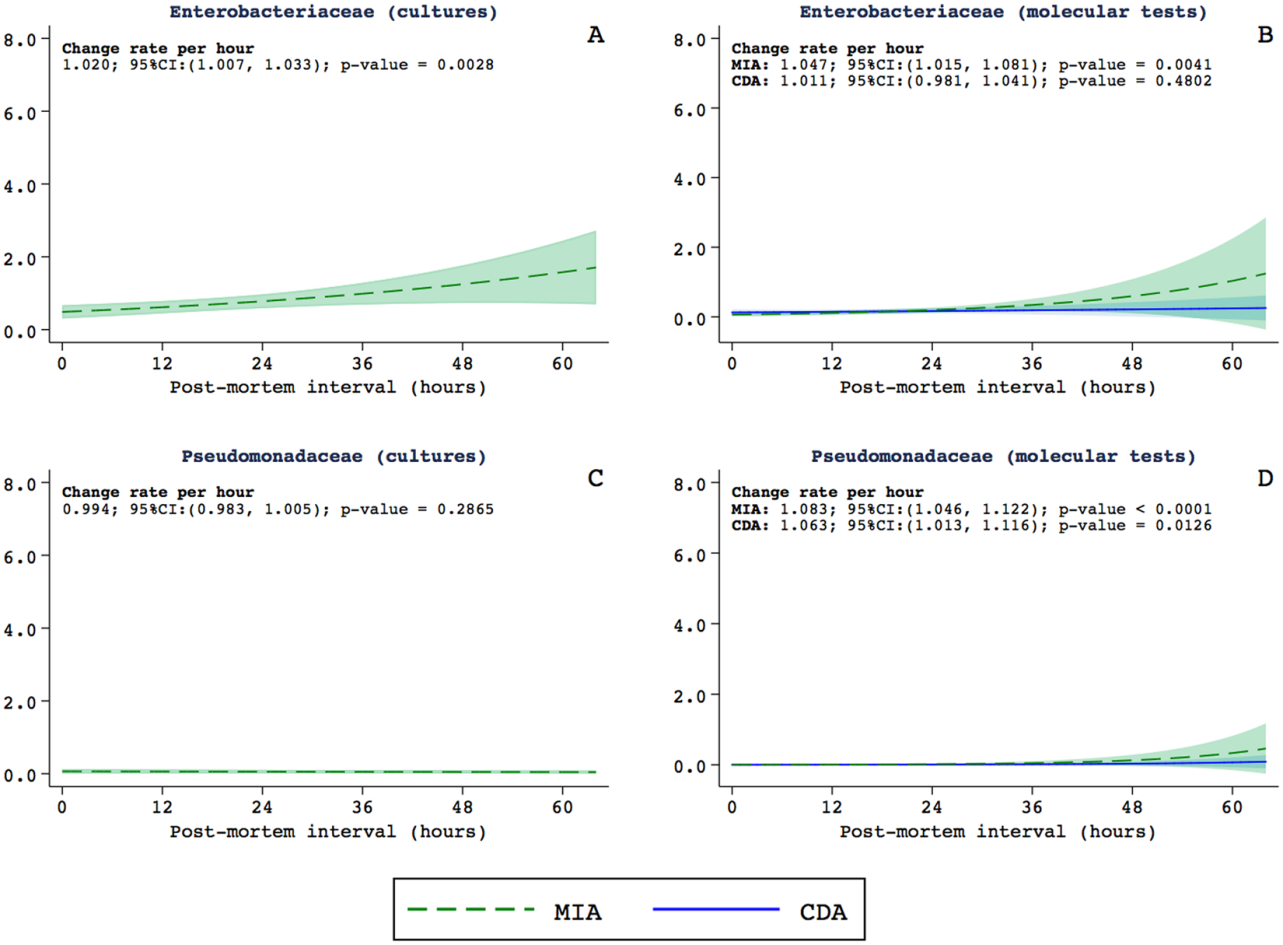
Relationship between the postmortem interval and the estimated detection rates of *Enterobacteriaceae* in cultures (**A**), and in molecular tests (**B**), *Pseudomonadaceae* in cultures (**C**), and in molecular tests (**D**). For molecular tests, the graphics include the estimations for the MIA (in green) and the CDA (in blue).

The analysis of the different families of bacteria showed that long PMI were associated with high rates of the identification of *Enterobacteriaceae* in the MIA samples both in conventional cultures (Fig. 3A) and in molecular tests (Fig. 3B). This association was not observed in the CDA samples. Increases in the PMI were associated with increases in the identification of *Pseudomonas genus* both in the MIA and in the CDA samples in the molecular analyses, but not in the conventional cultures (Fig. 3C,D).

There were borderline significant differences in the number of patients considered to have died of infections caused by strains of *Enterobacteriaceae* family or *Pseudomonas* genus between the early and the late autopsy groups (15/214 [7.0%] vs. 10/68 [14.7%] (p = 0.0518). When adding to these patients, the cases in which these bacteria were considered as having contributed to the death (addition of infections included in the chain of events leading to death, but not directly having caused the death and the infections considered as other significant conditions) the differences became significant with less deaths attributed to these bacteria in the early autopsy group (19/214 [8.9%]) than in the late autopsy group (13/68 [19.1%]) (p = 0.0204).

Finally, in only one of the 15 patients (6.7%) of the early autopsy group and in 2/10 (20.0%) of the late autopsy group, who were considered to have died of infections caused by strains of the *Enterobacteriaceae* family or *Pseudomonas* genus, the final diagnosis was established based on very low pathological evidence and, consequently it was mainly based on the microbiological evidence.

## Discussion

The appearance of the minimally invasive autopsy for post-mortem sampling to investigate the causes of death has stirred a significant revolution in the way we study mortality in low-income settings. However, short timeframes after death may hamper the feasibility of the MIA procedure when implementing the tool in low and middle-income countries, particularly in health facilities located in rural areas. For this reason, it appears relevant to generate evidence about the robustness of the data provided through the MIA beyond the first 24 hours after death.

In our study, no variations were observed related to the PMI in the number of cases in which no final diagnosis of cause of death was reached between the MIA and the CDA. Moreover, no diagnostic differences were observed between procedures performed earlier and later than 24 hours after death^3–6^. No differences were observed either regarding the level of between the MIA and the CDA diagnosis between the early and the late autopsy groups and no variations were observed either for the non-infectious conditions or for most of the infectious diseases. In particular, the microbiological results related to fungi and viruses seem not to be affected by changes related to the PMI and are, consequently, reliable^23,24^. These findings indicate that the MIA and the CDA, performed more than 24 hours after death can yield reliable results, and support their use after this period.

However, our study showed raising numbers of bacteria identified in the MIA (rate: 1.014 per hour [95%CI: 1.002–1.026]; p = 0.0228) with increasing PMI. This raise in the number of identified microorganisms with increasing PMI was evident for contaminating bacteria, but an increase was also observed in the number of bacteria considered as associated with true infections according to our criteria. This increase was mainly related with increases in the number of *Enterobacteriaceae* family and *Pseudomonas* genus strains.

In our study, the classification of a microorganism as a contaminant or as a microorganism associated with true infection was largely based on a grading scheme, which depend on the internal validity of the results obtained from the microbiology tests^3–6^. This grading scheme to determine the level of strength of a particular finding was applied not only to the microbiological results, but also to the pathological findings. For the microbiological findings, these criteria were based on the pathogenicity of each individual microorganism, but also on the number of organs (one or more organs) and the type of techniques (classic cultures, molecular tests or both) in which the microorganism was identified^3–6^. Accordingly, whereas some microorganisms such as *Cryptococcus* spp. or *M*. *tuberculosis* were always considered as associated with true infections, other microorganisms such as *Enterobacteriaceae* could be considered as contaminants or as associated with true infections depending on whether they have been isolated in a single or in multiple organs and on whether they had been identified by cultures, molecular tests or by both techniques simultaneously. However, these criteria are obviously influenced, not only by the sensitivity and specificity of the tests, but also by the quality of the samples.

The results of this study indicate that the abovementioned criteria may overestimate the contribution of *Enterobacteriaceae* and *Pseudomonas* spp. as responsible of infections leading or contributing to death, particularly in procedures performed more than 24 hours after death. The higher number of deaths attributed to these microorganisms in the late autopsy group (10/68 [14.7%] vs. 15/214 [7.0%] in the early autopsy group, p = 0.0518), and of infections contributing to death (13/68 [19.1%] in the late autopsy group vs. 19/214 [8.9%] in the early autopsy group, p = 0.0204) are in keeping with this hypothesis. However, the number of cases in which the diagnosis was established based only on the microbiological criteria, which might represent true over diagnosis, was very small (3 cases) whereas most of the cases had strong pathological evidence and represented only an overestimation of the etiological agent.

Bacterial translocation is a physiologic phenomenon that occurs during life and after death^15^. Viable bacteria originating principally from the intestinal microbiota have been isolated in samples (such as blood) taken from human bodies, providing evidence that this bacterial translocation occurs before death^16^. The three main factors resulting in this phenomenon in the living human (intestinal mucosa alteration, modification of the intestinal microbiota, and a weak immune system) also occur after death^25^. However, the results reported in the literature about the relationship between the PMI and detection of microorganisms are diverse. There is some controversy regarding the effect of the PMI on bacterial isolation rates. It has been reported a small effect of the PMI on the isolation rates in cultures from blood and CSF^13^; in contrast, other studies have shown that the PMI have no influence on bacterial growth^26^. However, in the latter series no sample was taken beyond 48 hours and only pure growth of potentially pathogenic bacteria were included in the analysis. Published data showing that *Escherichia coli*, *Klebsiella pneumoniae*, *Clostridium* spp, *P*. *aeruginosa*, *Enterococci*, and *Streptococci* have a higher potential of translocation^27,28^, are in keeping with our findings and stress the need of considering the PMI when evaluating the microbiological results related to these bacteria. Finally, a longer PMI was associated with an increase in the number of *Pseudomonas* spp. strains detected with molecular techniques. In contrast, an increased PMI was not associated with an increase in the number of strains of this genus in conventional cultures. This may be related to the low number of translocated bacteria, which can be detected by molecular tools but not by culture, because anaerobic strains limit the growth of species with high translocation potential^28^.

In our study, the bodies were refrigerated between the time of death and the autopsy procedures. However, some delays in the refrigeration with bodies exposed to high temperatures cannot be excluded. Refrigeration has been shown to reduce autolysis^29,30^ and bacterial translocation^13,31^, and consequently expand the period in which the postmortem pathological and microbiological analyses are reliable. Inaccessibility to refrigeration may reduce the efficiency interval and thus hinder the performance of the postmortem examination.

In conclusion, the MIA and the CDA, performed more than 24 hours after death can yield reliable results, provided bodies have been kept under adequate refrigeration. However, the contribution to death of *Enterobacteriaceae* and *Pseudomonas* spp. infections may be overestimated, particularly in procedures performed more than 24 hours after death. Thus, these bacteria should be probably disregarded as cause or contributors to death when evaluating autopsy procedures performed more than 24 hours after death. Further studies are needed to determine the maximum time beyond which such investigations using pathological and microbiological methods are likely to no longer provide valuable results.

## Electronic supplementary material

Supplementary tables

## Data Availability

All relevant data are within the paper. Any additional data use and transfer is monitored by ISGlobal’s Data Management and Biostatistics Unit (contact e-mail: ubioesdm@isglobal.org).
