# Supplementary material for: Postmortem Interval and Diagnostic Performance of the Autopsy Methods
Source: Sci Rep. 2018 Oct 31;8:16112. doi: 10.1038/s41598-018-34436-1 (PMC6208334; doi:10.1038/s41598-018-34436-1)
Supplement: Supplementary file 1 — Supplementary tables [file 41598_2018_34436_MOESM1_ESM.docx]

**“Postmortem Interval and Diagnostic Performance of the Autopsy Methods”**

Juan Carlos Hurtado^1,2,&^, Llorenç Quintó^1,&^, Paola Castillo^1,3,&^, Carla Carrilho^4,5^, Fabiola Fernandes^4,5^, Dercio Jordao^4^, Lucilia Lovane^4^, Mireia Navarro^1,2^, Isaac Casas^1,2^, Rosa Bene^6^, Tacilta Nhampossa^7^, Paula Santos Ritchie^8^, Sónia Bandeira^8^, Calvino Sambo^8^, Valeria Chicamba^8^, Sibone Mocumbi^9^, Zara Jaze^9^, Flora Mabota^9^, Mamudo R. Ismail^4,5^, Cesaltina Lorenzoni^4,5^, Assucena Guisseve^4,5^, Natalia Rakislova^1,3^, Lorena Marimon^1,3^, Natalia Castrejon^3^, Ariadna Sanz^2^, Anelsio Cossa^7^, Inacio Mandomando^7^, Khátia Munguambe^5,7^, Maria Maixenchs^1,7^, Carmen Muñoz-Almagro^10,11,12^, Eusebio Macete^7^, Pedro Alonso^1,7^, Jordi Vila^1,2^, Quique Bassat^1,7,13,14^, Clara Menéndez^1,7,11^, Miguel J. Martínez^1,2,^**^#^**, Jaume Ordi^1,3,^**^#^**^,^*****

***Address for correspondence:** jordi@clinic.ub.es

1. *ISGlobal, Hospital Clinic - Universitat de Barcelona, Barcelona, Spain*
2. *Department of Microbiology, Hospital Clinic - Universitat de Barcelona, Barcelona, Spain*
3. *Department of Pathology, Hospital Clinic - Universitat de Barcelona, Barcelona, Spain*
4. *Department of Pathology, Maputo Central Hospital, Maputo, Mozambique*
5. *Faculty of Medicine, Eduardo Mondlane University, Maputo, Mozambique*
6. *Department of Medicine, Maputo Central Hospital, Maputo, Mozambique*
7. *Centro de Investigação em Saúde de Manhiça, Maputo, Mozambique*
8. *Department of Pediatrics, Maputo Central Hospital, Maputo, Mozambique*
9. *Department of Gynecology and Obstetrics, Maputo Central Hospital, Maputo, Mozambique*
10. *Department of Molecular Microbiology, University Hospital Sant Joan de Déu (University of Barcelona), Barcelona, Spain*
11. *Consorcio de Investigación Biomédica en Red de Epidemiología y Salud Pública (CIBERESP), Spain*
12. *Faculty of Medicine, Univesitat Internacional de Catalunya, Barcelona, Spain*
13. *ICREA, Catalan Institution for Research and Advanced Studies, Pg. Lluís Companys 23, 08010 Barcelona, Spain*
14. *Pediatric Infectious Diseases Unit, Pediatrics Department, Hospital Sant Joan de Déu (University of Barcelona), Barcelona, Spain*

***^&^*** *These authors equally contributed* *to the work, and should share co-primary* *authorship.*

***^#^*** *These authors equally contributed* *to the work, and should share co-senior* *authorship.*

**Supplementary Table 1**. Level of strength of the pathological and microbiological findings of the CDA and the MIA between early and late autopsy groups.

| Variable | Time from death to MIA | | Total (N=282) | p-value |
| --- | --- | --- | --- | --- |
|  | ≤ 24h (N=214) | > 24h (N=68) |  |  |
| CDA | | | | |
| Level of strength (Pathology) ^1^ | 3.6; (3.5, 3.7) | 3.3; (3.1, 3.6) | 3.6; (3.4, 3.7) | 0.0155 ^2^ |
| Level of strength (Microbiology) ^1^ | 2.3; (2.1, 2.6) | 2.4; (2.0, 2.8) | 2.4; (2.2, 2.6) | 0.6888 ^2^ |
| Difference in level of strength (Pathology - Microbiology) ^1^ | 1.3; (1.1, 1.5) | 0.9; (0.4, 1.4) | 1.2; (1.0, 1.4) | 0.1182 ^2^ |
| MIA | | | | |
| Level of strength (Pathology) ^1^ | 2.7; (2.5, 2.9) | 2.4; (2.0, 2.8) | 2.6; (2.4, 2.8) | 0.1749 ^2^ |
| Level of strength (Microbiology) ^1^ | 2.2; (2.0, 2.4) | 2.4; (2.0, 2.8) | 2.2; (2.0, 2.4) | 0.4460 ^2^ |
| Difference in level of strength (Pathology - Microbiology) ^1^ | 0.5; (0.2, 0.7) | 0.0; (-0.5, 0.5) | 0.4; (0.1, 0.6) | 0.0816 ^2^ |
| Difference CDA - MIA | | | | |
| Level of strength (Pathology) ^1^ | 0.9; (0.7, 1.1) | 0.9; (0.6, 1.3) | 0.9; (0.8, 1.1) | 0.9480 ^2^ |
| Level of strength (Microbiology) ^1^ | 0.1; (-0.1, 0.3) | 0.0; (-0.2, 0.3) | 0.1; (-0.1, 0.3) | 0.6716 ^2^ |
| Difference in difference (Pathology - Microbiology) in level of strength ^1^ | 0.8; (0.5, 1.1) | 0.9; (0.4, 1.4) | 0.8; (0.6, 1.1) | 0.8022 ^2^ |

1: Arithmetic Mean; (95% Conf. Interval) 
2: t-test

**Supplementary Table 2.** Microorganisms identified in the study. In bold, microorganisms categorized as having caused disease in some of the cases based on previously reported criteria [Castillo, P. et al. Validity of a minimally invasive autopsy for cause of death determination in adults in Mozambique: an observational study. PLoS Med. 13, 10.1371/journal.pmed.1002171 (2016); Bassat, Q. et al. Validity of a minimally invasive autopsy tool for cause of death determination in pediatric deaths in Mozambique: An observational study. PLoS Med. 14, 10.1371/journal.pmed.1002317 (2017), and Menendez, C. et al. Validity of a minimally invasive autopsy for cause of death determination in stillborn babies and neonates in Mozambique: An observational study. PLoS Med. 14, 10.1371/journal.pmed.1002318 (2017)]

| **Microorganisms** |
| --- |
| ***Acinetobacter baumannii*** |
| *Acinetobacter baylyi* |
| *Acinetobacter guillouiae* |
| *Acinetobacter johnsonii* |
| *Acinetobacter lwoffii* |
| *Acinetobacter pittii* |
| ***Acinetobacter spp.*** |
| **Adenovirus** |
| *Aerobacillus spp.* |
| *Aeromona sobria* |
| ***Aeromonas caviae*** |
| ***Aeromonas hydrophila*** |
| ***Aeromonas jandaei*** |
| *Aeromonas punctate* |
| ***Aeromonas sobria*** |
| ***Aeromonas spp.*** |
| ***Aeromonas veronii*** |
| *Alcaligenes faecalis* |
| *Alkalimonas spp.* |
| *Arthrobacter spp.* |
| *Bacillus cereus* |
| *Bacillus licheniformis* |
| *Bacillus megaterium* |
| *Bacillus spp.* |
| *Bacteroides fragilis* |
| *Bacteroides vulgatus* |
| ***Burkholderia cepacia*** |
| *Burkholderia spp.* |
| ***Candida albicans*** |
| ***Candida glabrata*** |
| *Candida parapsilosis* |
| *Candida spp.* |
| *Candida tropicalis* |
| *Chitinophaga spp.* |
| *Chryseobacterium indologenes* |
| *Chryseobacterium spp.* |
| ***Citrobacter braakii*** |
| *Citrobacter freundii* |
| *Cladosporium sphaerospermum* |
| *Clostridium botulinum* |
| *Clostridium limosum* |
| *Clostridium novyi* |
| *Clostridium perfringens* |
| *Clostridium sordellii* |
| *Clostridium spp.* |
| Coagulase-Negative Staphylococci |
| *Comamonas aquatic* |
| *Comamonas terrigena* |
| *Corynebacterium accolens* |
| *Corynebacterium pseudodiphteritium* |
| *Corynebacterium spp.* |
| *Cronobacter sakazakii* |
| ***Cryptococcus gattii*** |
| ***Cryptococcus neoformans*** |
| *Curvularia hawaiiensis* |
| **Cytomegalovirus** |
| *Dolosigranulum pigrum* |
| *Dothideomycetes spp.* |
| *Endophytic bacterium* |
| ***Enterobacter aerogenes*** |
| *Enterobacter amnigenus* |
| *Enterobacter asburiae* |
| ***Enterobacter cloacae*** |
| *Enterobacter hormaechei* |
| *Enterobacter kobei* |
| *Enterobacter sakazakii* |
| ***Enterobacter spp.*** |
| *Enterococcus faecalis* |
| *Enterococcus spp.* |
| ***Escherichia coli*** |
| *Escherichia fergusonii* |
| *Escherichia spp.* |
| *Eubacterium tarantellae* |
| *Fusarium merismoides* |
| *Fusarium spp.* |
| *Fusobacterium gonidoformans* |
| *Fusobacterium nucleatum* |
| *Fusobacterium spp.* |
| ***Haemophilus influenzae*** |
| *Haemophilus parainfluenzae* |
| **Hepatitis B Virus (HBV)** |
| **Human Immunodeficiency Virus (HIV)** |
| **Herpes Simplex Virus type 1 (HSV-1)** |
| **Herpes Simplex Virus type 2 (HSV-2)** |
| *Klebsiella oxytoca* |
| ***Klebsiella pneumoniae*** |
| *Klebsiella spp.* |
| *Klebsiella variicola* |
| *Kluyvera spp.* |
| *Lactobacillus crispatus* |
| *Lactobacillus fermentum* |
| ***Lactobacillus reuteri*** |
| *Lactobacillus spp.* |
| *Leclercia adecarboxylata* |
| ***Legionella pneumophila*** |
| ***Moraxella catarrhalis*** |
| *Moraxella osloensis* |
| *Moraxella spp.* |
| *Morganella morganii* |
| ***Mycobacterium tuberculosis*** |
| ***Mycoplasma hominis*** |
| ***Mycoplasma spp.*** |
| *Neisseria flavescens* |
| *Nigrospora oryzae* |
| *Ochrobactrum anthropic* |
| *Ochroconis constricta* |
| *Pantoea agglomerans* |
| *Pantoea dispersa* |
| *Pantoea spp.* |
| *Paracoccus spp.* |
| Parvovirus B19 |
| *Pasteurella multocida* |
| *Penicillium chrysogenum* |
| *Penicillium spp.* |
| *Phanerochaete chrysosporium* |
| *Phoma spp.* |
| ***Pneumocystis jirovecii*** |
| *Porphyromonas spp.* |
| *Prevotella histolytica* |
| ***Prevotella melaninogenica*** |
| ***Prevotella oris*** |
| *Prevotella ruminicola* |
| ***Prevotella spp.*** |
| *Prevotella stercorea* |
| *Prevotella veroralis* |
| ***Proteus mirabilis*** |
| *Proteus penneri* |
| *Proteus spp.* |
| ***Providencia alcalifaciens*** |
| *Providencia spp.* |
| *Providencia stuartii* |
| ***Pseudomonas aeruginosa*** |
| *Pseudomonas fluorescens* |
| *Pseudomonas fulva* |
| *Pseudomonas lundensis* |
| *Pseudomonas monteilii* |
| *Pseudomonas parafulva* |
| *Pseudomonas spp.* |
| *Pseudomonas stutzeri* |
| *Pseudomonas synxantha* |
| *Raoultella ornithinolytica* |
| *Raoultella terrigena* |
| ***Rhinovirus*** |
| ***Rhizopus oryzae*** |
| *Rhodotorula mucilaginosa* |
| *Saccharomyces cerevisiae* |
| ***Salmonella enterica*** |
| ***Salmonella tiphy*** |
| ***Salmonella spp.*** |
| *Serratia ficaria* |
| *Serratia liquefaciens* |
| ***Serratia marcescens*** |
| *Shewanella putrefaciens* |
| *Sneathia amni* |
| *Sneathia sanguinegens* |
| *Sphingomonas spp.* |
| ***Staphylococcus aureus*** |
| *Staphylococcus epidermidis* |
| *Staphylococcus hominis* |
| *Stenotrophomonas maltophilia* |
| ***Streptococcus agalactiae*** |
| *Streptococcus anginosus* |
| ***Streptococcus dysgalactiae*** |
| *Streptococcus gallolyticus* |
| *Streptococcus* Group C |
| *Streptococcus* Group G |
| *Streptococcus oralis* |
| ***Streptococcus pneumoniae*** |
| *Streptococcus pseudopneumoniae* |
| ***Streptococcus pyogenes*** |
| ***Streptococcus spp.*** |
| *Streptococcus viridans* |
| ***Toxoplasma gondii*** |
| *Trichosporon jirovecii* |
| *Trichosporon montevideense* |
| ***Tropheryma whipplei*** |
| *Veillonella parvula* |
| *Vibrio vulnificus* |
| **Parainfluenza Virus type 1 (PIV-1)** |
| **Parainfluenza Virus type 3 (PIV-3)** |
| **Parainfluenza Virus type 4 (PIV-4)** |
| **Respiratory Syncytial Virus** |
| *Weissella confuse* |
| *Xanthomonas citri* |
